# Targeting low-grade inflammation in multiple sclerosis through the Wim Hof method or lifestyle intervention: a pilot comparative study

**DOI:** 10.1007/s10072-026-08895-8

**Published:** 2026-02-26

**Authors:** Darina Slezáková, Louise Mária Sabolová, Peter Marček, Pavol Kadlic, Ivan Hric, Libuša Nechalová, Viktor Bielik, Michal Páleník, Michal Pastorek, Peter Olej, Norbert Žilka, Jozef Hanes, František Jurčaga, Michal Minár

**Affiliations:** 1 Second Department of Neurology, Faculty of Medicine, Comenius University, University Hospital in Bratislava, Bratislava, Slovakia; 2 Biomedical Research Center, Institute of Clinical and Translational Research, Slovak Academy of Sciences, Bratislava, Slovakia; 3 Department of Biological and Medical Science, Faculty of Physical Education and Sport, Comenius University in Bratislava, Bratislava, Slovakia; 4 Institute of Molecular Biology, Faculty of Natural Sciences, Comenius University in Bratislava, Bratislava, Slovakia; 5 Department of Gymnastics, Faculty of Physical Education and Sport, Comenius University in Bratislava, Bratislava, Slovakia; 6 Institute of Neuroimmunology, Slovak Academy of Sciences, Dúbravská Cesta 9, Bratislava, Slovak Republic; 7 Neuroimmunology Institute, Dvořákovo nábrežie 7527/10, Bratislava, 811 02 Slovak Republic; 8 Department of Neurology, St. Michal University Hospital, Bratislava, Slovak Republic

**Keywords:** Multiple sclerosis, Wim Hof method, Lifestyle modification, Excercise, Inflammation, Non-pharmacological therapy

## Abstract

**Background:**

Disease-modifying therapies (DMTs) in multiple sclerosis (MS) effectively reduce relapse activity but have limited impact on chronic progression and neurodegeneration. Non-pharmacological interventions such as structured exercise or the Wim Hof Method (WHM- which includes breathing exercises, cold exposure, and meditation), may offer complementary immunomodulatory and neuroprotective benefits.

**Objectives:**

To compare the effects of WHM and a lifestyle intervention (LIFE; structured physical activity and nutritional counseling) on systemic inflammation and neurodegeneration biomarkers in patients with MS.

**Methods:**

In this randomized, prospective pilot trial, 60 MS patients (2017 McDonald criteria, EDSS 1.0–5.5) were allocated to WHM, LIFE, or control (CTRL) for 12 weeks. Serum cytokines (IFN-γ, IL-1β, IL-6, IL-8, IL-10, IL-12p70, IL-17A, IL-18), neurofilament light chain (NfL), and glial fibrillary acidic protein (GFAP) were assessed at baseline and after 12 weeks. Mixed repeated-measures ANOVA with Bonferroni correction was applied.

**Results:**

Complete datasets were obtained and analyzed for 43 participants (12 WHM, 17 LIFE, 14 CTRL; power= 0.81). Both interventions significantly reduced IL-17A and IL-18 (p<0.001), indicating attenuation of Th17-related inflammation. WHM further decreased IFN-γ, while LIFE lowered IL-8. No significant changes were observed for IL-1β, IL-6, IL-12p70, NfL, or GFAP. Both interventions were well tolerated, with no treatment-related adverse events.

**Conclusions:**

Both WHM and lifestyle modification demonstrated comparable short-term anti-inflammatory effects in MS, supporting their safety and feasibility as adjunctive strategies to DMT. Although neurodegeneration biomarkers remained unchanged, the consistent reduction of IL-17A and IL-18 highlights their potential to modulate smoldering inflammation. Larger, longer-term trials are warranted to determine their sustained effects on disease progression.

## Introduction

Smoldering multiple sclerosis (MS), with limited pharmacological options for treating chronic neuroinflammation and progression independent of relapse activity (PIRA), has sparked growing interest in adjunct interventions targeting environmental factors of disease pathogenesis [1, 2]. This unmet therapeutic need underscores the importance of complementary non-pharmacological strategies aimed at modulating inflammation beyond acute relapses [3]. Current research focuses on exercise and dietary strategies as accessible non-pharmacological therapies that may slow progression and improve quality of life [4, 5].

Regular physical activity benefits MS patients across disease stages by improving physical function, reducing fatigue, enhancing quality of life, and potentially slowing progression. These effects likely stem from immunomodulatory, anti-inflammatory, and neuroprotective mechanisms supported by imaging and biomarker data [4–7]. However, most studies are short-term (1–6 months) and limited by poor patient adherence and the absence of long-term follow-up, which restricts the ability to detect changes in brain volume or disease progression. Consequently, the definitive immunomodulatory effect of exercise remains unproven [8–13].

Previous research has shown that the immune system is partially modulated also by the autonomic nervous system (ANS) by increasing vagal tone. Short training based on hyperventilation with breath retention, cold exposure, and meditation - the so-called Wim Hof Method (WHM) – proved to reduce proinflammatory cytokine response experimentally induced by intravenous administration of bacterial endotoxin. A decrease in the levels of TNF-α, IL-6, and IL-8 was accompanied by the release of epinephrine and elevation of the immunosuppressive hormone cortisol [14, 15]. Buijze et al. applied WHM to patients with axial spondylarthritis, resulting in a subsequent decrease in acute inflammatory activity [16], but overall evidence of WHM effect on various autoimmune diseases is scarce.

We present a pilot comparative trial to evaluate whether WHM and lifestyle intervention may exert immunomodulatory and neuroprotective effects in MS, focusing on their potential role in modulating chronic inflammation and disease progression.

## Methods

### Subjects

In this randomized prospective interventional study, we included 60 patients with.

- MS diagnosed according to 2017 McDonald criteria,
- age 18–55 years,
- EDSS 1.0-5.5,
- disease duration of at least 1 year.

Exclusion criteria were serious comorbidities, and/or recent relapse (less than 3 months), including any worsening that required corticosteroids.

Informed consent was obtained from all participants. The research was approved by the Ethics Committee of Derer's University Hospital Bratislava (No. 27/2022).

Patients were randomly assigned to:

1. the Wim Hof Method (WHM) Group - a 12-week supplementary training program incorporating supervised cold exposure, breathing exercises, and mindfulness,
2. the Lifestyle Intervention (LIFE) Group a 12-week supervised physical activity (dance classes) [17] and nutritional intervention program (reduced caloric intake), and.
3. the Control (CTRL) Group consisted of patients without any intervention.

### Wim Hof method

Each full meeting was persuaded weekly on Saturdays under the supervision of certified trainer and took place in silent training room.

At the commencement of the meeting, we gave participants an introduction to breathing technique, and explained how it affects autonomic nervous system by repeated hyperventilation and holding of breath according to Wim Hof. The total duration of the breathing exercise was 40 to 55 min. Participants were lying down comfortably in a relaxed position. Each breathing cycle consisted of approximately 30 deep breaths. Participants were guided to exhale, hold the breath with empty lungs, then inhale deeply and hold their breath for approximately 15 s. We managed to make from 4 to 7 breathing cycles during each meeting. While breathing, we regularly trained their imagination and visualization by setting their attention to different body parts and internal organs, so we were purposefully connecting the brain with various parts of their body. Participants stretched and relaxed their whole body, each one according to its needs. During the breathing exercise we used the effects of music therapy. The musician was playing various musical instruments, such as Tibet bowls, kalimbas, shamanic drums, kachon, drumbles and rattles. The sound waves were spread in the environment and directly above each patient so they could closely feel the entire sound wave.

After the breathing warm up, participants put on their swimsuits and went straight to the cold river located 50 m from the building where the breathing exercises took place. All of them at once, along with the trainer, carefully entered the water keeping their head above the water surface for 2 to 3 min. The temperature of the water was 6 to 7 degrees Celsius.

Right after they exit the water, without wiping their body, they practiced the horse stance from Chi Kung recommended by Wim Hoff to warm up the body with movement. In this form of the stance, the knees are bent slightly so that they are directly above the toes. The arms are raised slowly to waist level with the elbows kept close to the torso, which leans forward slightly. The shoulders and back muscles are completely relaxed throughout the exercise. The feet are placed firmly on the ground about shoulder-width apart. The practice was performed for 20 min.

Afterwards, they went straight inside to the training room again and more moves like shaking, jumping and dancing were incorporated.

Beyond that, an individual cold exposure program was created. Every weekday participants started by 3 to 4 rounds of guided breathing exercises according to Wim Hof, explained above, with duration of 11 min. The type of hardening session during the week varied so that they did not have to have a cold dip every day. Two weekdays they did morning cold shower for 1–2 min. They were guided to concentrate on muscle relaxation starting from the feet, while they had to breathe slowly through their noses and control their muscles with their minds. After the shower, they did dance moves or horse stance exercise. Another two weekdays they put their hands and feet to the sink with cold, warm or air temperature water. To gradually increase the cold exposure, the length of the exposure started at 10 s and was increased to a maximum of 2 min. They did at least 3 rounds. On these days, they should start the morning with breathing exercises and then practice some short sequence of exercises from yoga, Tai Chi, Chi Kung, Pilates, or play their favorite music and dance. One day a week they were exposed to the temperatures outside, either in summer clothes or swimwear. They could either stand there and breathe freely, do moderate physical exercise, roll around in the snow, or take a short walk in light clothes. These cold exposures usually lasted from 5 to 15 min. On Sundays they did only longer breathing exercises at home, accompanied by relaxing music.

From the second week, pictures and videos of internal organs and body systems were added to the training sessions. Participants were guided to look at them for 5 min every day, so that they could recall in their minds how they look like. From the fourth week, visualization of different parts of the body while breathing was incorporated to the program. They were instructed to imagine the brain, the nerve cells, and their projections, how they connect with each cell and how the electrical discharge passes from the nerve cell to different parts of the body. From the fourth week, heat visualization during the warm-up was added. At least twice a week, they should imagine heat creation when heating, using all their senses. It means imagining an image that visually represents heat for them, the feeling on the skin when they feel heat, the smell of heat, or the taste of a warm drink or food.

The training sessions alternated so that they always had something more difficult one weekday and less difficult the next day. They could stop session at any time or replace it with a less difficult one if they felt they needed a break.

Both group and individual meetings were accustomed to patients’ functional limitations and specific needs.

### Physical exercise

Rock and roll classes were persuaded weekly on Tuesdays, and sports dances on Thursdays under supervision of certificated trainers. The goal of rock and roll classes was to learn and develop the basic skills and techniques of rock and roll. At the commencement of the program, we initiated the exercise routine with a total of eight repetitions, divided into two sets for each step. Basic steps were gradually combined with the dance figures. Initially, the movement was executed at a slow pace, devoid of musical accompaniment and lacking in rhythmic buoyancy. Subsequently, the tempo increased, accompanied by a rhythmic bounce and the addition of musical elements. Once the correct technique was established, we proceeded to integrate individual steps into different combinations, which were subsequently incorporated into each practice session of the program as a designated warm-up routine. The tempo of the music utilized during training sessions varied between 156 and 184 beats per minute (bpm), with the specific value being contingent upon the level of difficulty associated with the exercises being performed. Throughout the duration of the program, a total of five choreographies were constructed, each with a varying duration ranging from 30 to 60 s.

Sports dance classes on Thursdays aimed to teach the basics of ballroom dances (waltz, tango, Viennese waltz, quickstep) and Latin American dances (samba, chacha, rumba, jive). Both types of dance classes started with a 5-minute warm-up, followed by 10-minute dynamic stretching, and then the 40-minute main part, which we always ended with static stretching. The patients became familiar with the various dances, as well as the traits, beats, and fundamental figures that we blended together to create basic sets. Ten counts were made of each new step, followed by five counts while listening to calm music. After that, more moves were incorporated and linked to the choreography, which was performed five times: five times at a slow tempo, five times at a standard tempo, and five times while counting. The music’s tempo was adjusted to fit their physical state at the time. The waltz, which has the slowest pace, opened the program, and the jive, which has one of the fastest tempos, closed it. Every training unit also included a repetition of previously taught dances. Each dance lasted one minute and was performed five times.

Every subject wears a heart rate chest belt synchronized with a portable Polar Team Pro (Polar Electro, Kempele, Finland) to monitor the intensity of training.

### Diet

The patients in exercise group were required to follow a 12-week weight loss diet that included reduced caloric intake. Each patient received detailed instructions and counseling about lifestyle changes, as well as a personalised nutritional plan created with the software PLANEAT (www.planeat.sk; accessed on September 6, 2014). The daily calorie intake was made up of 45% carbohydrates, 30% fat, and 25% protein. For feedback and control, all participants were asked to report their food consumption and 3 times during intervention was organized an on-line discussion (MS Teams) for motivation and socialization purpose. Planeat nutrition software (Planeat s.r.o., Bratislava, Slovakia) was used to analyze quantitative and qualitative data. All the participants’ food reports were collected and analyzed, and one random week was analyzed and averaged.

## Data collection

We collected basic demographic data (age, sex, disease duration, EDSS score, type of DMT); any adverse events and adherence was monitored throughout the whole trial duration.

Blood serum and plasma was assessed for markers of inflammation and neurodegeneration at enrollment and after 12 weeks.

The concentrations of cytokines (IFN-γ, IL-1β, IL-6, IL-8, IL-10, IL-12p70, IL-17 A, IL-18) were determined using the LEGENDplex™ Human Inflammation Panel (13-plex) in V-bottom plates (Biolegend, San Diego, CA, USA) and measured on a DxFlex cytometer (Beckman Coulter, Brea, CA, USA) according to the manufacturer’s instructions.

Concentrations of Neurofilament light chain in plasma were measured by digital ELISA using the Simoa^®^ NF-Light v2 Advantage Kit (Quanterix, Cat. No. 104073). GFAP was quantified using the Simoa^®^ GFAP Discovery Kit (Quanterix, Cat. No. 102336). The samples were blinded prior to analysis and both assays were measured on an HD-X Analyzer (Quanterix Corp, MA, USA). Concentrations were calculated using Simoa HD-X instrument software.

### Statistical analysis

The data were analyzed by JASP Team (2021) JASP (Version 0.16) software. Normality of residuals was assessed using Shapiro–Wilk tests, homogeneity of variance using Levene’s test, and potential outliers were screened using visual inspection of boxplots. When violations of sphericity were detected, Greenhouse–Geisser correction was applied.

Descriptive analyses were used to summarize demographic and clinical characteristics. Group comparisons were conducted using one-way ANOVA for normally distributed data, Kruskal-Wallis test for non-normally distributed data, and Chi-square test for categorical variables. Fisher’s exact test was used to compare dropout rates among groups due to small group sizes.

To assess the effect of intervention we used a mixed (between–within) repeated-measures ANOVA (we specifically tested the Time × Group interaction as the primary effect of interest - the effect of intervention over time). We considered p-value < 0.005 (after Bonferroni correction) to be significant. Significantly different parameters were *post hoc* analyzed comparing mean change (deltas of respective parameters) using ANOVA, p value < 0.05 was considered significant.

## Results

We collected and analyzed complete data from 43 patients (12 in WHM, 17 in LIFE, and 14 in CTRL group) which allowed us to perform statistical analysis with sufficient power of 0.81. Relevant demographic and clinical data are in Table 1. Groups showed no significant difference in age (*p* = 0.629), sex distribution (*p* = 0.302), disease duration (*p* = 0.327), or EDSS (*p* = 0.374) at baseline visit.

**Table 1:** Demographic and clinical data of WHM-, LIFE-, and CTRL-group of patients with MS who completed the study

Patients	WHM group	LIFE group	CTRL group	*p*
N	12	17	14	
Age (years; mean ± SD)	37.92 ± 8.273	42.81 ± 6.100	39.54 ± 8.891	0.629
Female sex (n/%)	6/50.0%	4/76.5%	10/71.4%	0.302
MS duration (years; mean ± SD)	5.58 ± 7.103	9.71 ± 6.165	8.61 ± 6.612	0.327
EDSS (median/IQR)	2.75/1.375	3.00/1.750	2.5/1.750	0.374
DMT (n/%)	11/91.7	16/94.1	12/85.7	0.720
Dimethyl fumarate	2	3	1	
Natalizumab	2	8	8	
Cladribine	2	1	1	
Ocrelizumab	1	3	0	
Rituximab	2	1	1	
Siponimod	1	0	0	
Fingolimod	1	0	1	
No DMT	1	1	2	

*MS* multiple sclerosis, *M *male, *F *female, *WHM *Wim Hof Method, *LIFE *lifestyle intervention group, *CTRL *control group, *EDSS *expanded disability status scale, *SD *standard deviation, *IQR *interquartile range

 Both interventions led to significant decrease of IL-17 A and IL-18. INF- γ significantly decreased in WHM group, IL-8 in LIFE group. There was no significant change in IL-1β, IL-6, and IL-12p70. There was no effect on levels of NfL and GFAP (Table 2).

**Table 2:** Serum cytokine and neurodegeneration marker levels at baseline and after 12-week intervention in MS patients

Parameter	Group	BL		EoS		F	*p*	n2	post-hoc ANOVA		*p*
		Mean	SD	Mean	SD						
NfL	CTRL	8.201	5.813	7.873	6.339	1.031	0.366	0.006			
	LIFE	8.121	2.428	8.640	3.168						
	WHM	7.636	3.166	6.677	2.428						
GFAP	CTRL	185.1	59.88	177.6	72.36	1.321	0.278	0.001			
	LIFE	211.7	75.56	180.5	91.66						
	WHM	196.7	72.66	185.1	99.27						
IFN-γ	CTRL	1.875	1.531	2.726	1.680	12.94	**< 0.001**	0.110	**CTRL**	**WHM**	**< 0.001**
	LIFE	2.401	1.594	0.457	0.354				CTRL	LIFE	0.687
	WHM	4.762	4.892	1.048	1.640				WHM	LIFE	< 0.006
IL-1β	CTRL	10.789	8.034	14.786	9.198	3.929	0.028	0.040			
	LIFE	14.219	11.865	7.978	7.359						
	WHM	21.281	24.866	7.963	8.843						
IL-6	CTRL	1.719	1.855	3.258	3.949	1.723	0.192	0.006			
	LIFE	4.548	9.067	3.303	4.452						
	WHM	2.659	2.097	2.033	1.315						
IL-8	CTRL	6.689	4.229	24.921	35.360	6.702	**0.003**	0.087	CTRL	WHM	0.080
	LIFE	11.896	11.584	3.018	2.863				**CTRL**	**LIFE**	**0.003**
	WHM	12.849	12.501	2.770	3.556				WHM	LIFE	0.509
IL-10	CTRL	2.421	2.578	6.401	10.265	4.610	0.016	0.055			
	LIFE	4.752	4.819	1.729	2.139						
	WHM	4.442	4.208	1.087	1.543						
IL-12	CTRL	1.132	1.245	2.327	3.288	3.796	0.031	0.033			
	LIFE	2.160	2.128	1.589	1.145						
	WHM	3.224	3.306	1.698	1.499						
IL-17	CTRL	0.356	0.358	0.577	0.630	8.543	**< 0.001**	0.077	**CTRL**	**WHM**	**0.044**
	LIFE	1.134	0.953	0.367	0.328				**CTRL**	**LIFE**	**< 0.001**
	WHM	0.725	0.831	0.232	0.301				WHM	LIFE	0.085
IL-18	CTRL	148.73	68.01	296.50	258.25	17.093	**< 0.001**	0.190	**CTRL**	**WHM**	**0.012**
	LIFE	140.59	65.56	53.10	36.67				**CTRL**	**LIFE**	**< 0.001**
	WHM	251.59	113.13	52.41	24.92				WHM	LIFE	0.475

*WHM *Wim Hof Method, *LIFE *lifestyle intervention group, *CTRL *control group

*BL *baseline; *EoS *end of study; *SD *standard deviation; *NfL *neurofilament light chain; *GFAP *glial fibrillary acidic protein; *IL *interleukin; *IFN-γ *interferon gamma

Cytokine concentrations are expressed in pg/mL; NfL and GFAP in ng/L

**Fig. 1:**
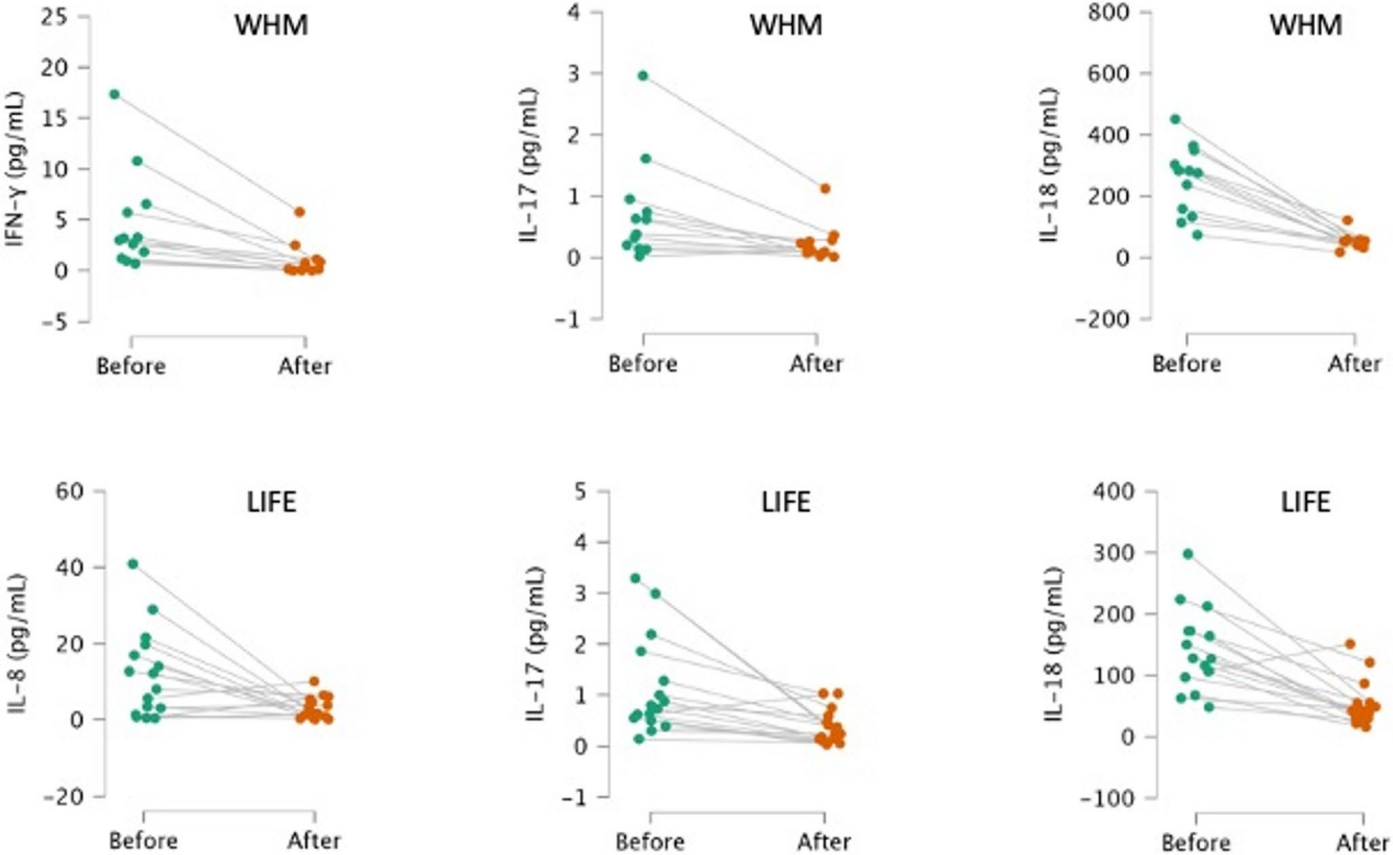
Significantly improved IFN-γ in WHM group (*p* < 0.001), IL-8 in LIFE group (*p* = 0.003), IL-17 A in both WHM (*p* = 0.044), and LIFE (*p* < 0.001), group, and IL-18 also in both WHM (*p* = 0.012), and LIFE (*p* < 0.001) group. (WHM – Wim Hof Method, LIFE – Lifestyle Intervention Group)

Significantly improved parameters are also presented in Fig. 1.

Although dropout rates appeared higher in the WHM group (40%, *n* = 8) compared to LIFE (15% *n* = 3) and CTRL (30% *n* = 6), this difference was not statistically significant (*p* = 0.366). There was no adverse event linked to either intervention recorded. All dropouts were due to patients’ decision to discontinue mostly because of subjectively complicated adherence to intervention and/or no willingness to continue.

## Discussion

Our randomized pilot trial demonstrated that both the Wim Hof Method (WHM) and a structured lifestyle program combining physical activity and nutritional counseling exert comparable short-term anti-inflammatory effects in relapsing-remitting multiple sclerosis (RRMS). Both interventions significantly reduced IL-17 A and IL-18, cytokines strongly implicated in chronic immune activation and progression independent of relapse activity (PIRA) [1–3]. WHM additionally decreased IFN-γ, while the lifestyle program selectively reduced IL-8, suggesting that these non-pharmacological strategies may modulate distinct but convergent immune pathways.

The observed cytokine shifts occurred without overt inflammation, indicating that both interventions primarily affected low-grade systemic immune activation rather than acute inflammatory responses. Such modulation aligns with the concept of smoldering MS, in which chronic innate and adaptive immune activation drives neurodegeneration even during clinical remission [1–3]. The control group exhibited minor, nonsignificant cytokine increases despite stable DMT use, likely reflecting physiological variability rather than loss of treatment efficacy.

Physical activity is established as a safe adjunct therapy in MS, capable of restoring balance between pro- and anti-inflammatory cytokines and enhancing neurotrophic signaling [1, 18–21]. Our results support its immunomodulatory impact but also highlight the time constraints of short-term interventions: neither NfL nor GFAP levels changed significantly within 12 weeks. However, only a limited number of studies have evaluated its impact on biomarkers of axonal and glial injury [12, 13]. Our findings align with the TREFAMS-AT study, where 16 weeks of aerobic training in patients with MS-related fatigue did not result in significant changes in serum NfL or GFAP [11]. Current evidence suggests that prolonged aerobic or combined training may reduce plasma NfL, particularly following moderate-intensity exercise, indicating a possible neuroprotective effect of physical activity [10, 12, 13]. The impact on GFAP remains inconsistent and requires standardized protocols [10–12]. This parallels prior human studies showing that longer or higher-intensity training is required to influence neurodegeneration markers [4, 5, 9]. Nevertheless, long-term training effects appear heterogeneous and depend on study design, exercise intensity, supervision, and timing of sampling [10].

WHM combines controlled breathing, cold exposure, and mindfulness to induce voluntary activation of the sympathetic nervous system and increased vagal tone, thereby modulating cytokine release and oxidative balance. Previous human trials confirmed its ability to attenuate endotoxin-induced inflammation [14–16] and to improve autonomic regulation in chronic inflammatory disorders [22]. To the best of our knowledge, no study to date has investigated the effects of the Wim Hof Method on inflammatory or neurodegenerative biomarkers in patients with multiple sclerosis. In our study, WHM reduced IFN-γ and IL-18, which is consistent with down-regulation of Th1-mediated responses. The absence of NfL or GFAP changes suggests that the neuroprotective effects of WHM, if present, require longer exposure or repeated training cycles. The focus of future studies should therefore shift from performance-oriented research in healthy individuals toward exploring its therapeutic potential in patients with inflammatory and neurodegenerative conditions such as MS.

Although their mechanisms differ—sympathetic-vagal modulation in WHM versus metabolic and neuromuscular adaptation in exercise—both converge on systemic inflammation control. The reduction of IL-17 A and IL-18 across groups underscores their shared ability to influence the Th17 axis, a recognized contributor to progressive tissue injury in MS. This finding is noteworthy, as the Th17 pathway, with IL-17 as its principal effector and IL-18 as a potent amplifier, plays a central role in the pathogenesis of autoimmune and inflammatory diseases, including MS [23]. Both cytokines are critically involved in neurodegenerative cascades through immune-mediated and direct neuronal mechanisms. Their interplay sustains chronic inflammation and promotes progressive neural injury, identifying them as promising therapeutic targets [23–25]. The observed down-regulation of IL-17 A and IL-18 in our study therefore suggests that WHM and structured lifestyle interventions might increase disease-modifying potential by simultaneously attenuating inflammatory and degenerative processes in MS.

Both interventions were well tolerated, with no adverse events reported. The higher dropout rate in the WHM group (40%) indicates that structured, individualized supervision is necessary to maintain adherence. Importantly, these approaches did not interfere with ongoing DMT, supporting their feasibility as safe adjuncts to pharmacological therapy. Integrating structured exercise or WHM-based autonomic training into MS management may complement DMT by addressing mechanisms of smoldering disease activity that remain pharmacoresistant. Their accessibility and low risk profile make them suitable for long-term lifestyle programs within multidisciplinary care frameworks.

### Limitations and future directions

Our study has several limitations. First, the 12-week duration and modest sample size limit the ability to detect long-term or subtle neuroprotective effects. Although statistical power was sufficient, larger and more homogeneous cohorts are required to confirm clinical efficacy. Second, when interpreting the observed decrease in pro-inflammatory biomarkers across both interventions—WHM and lifestyle modification including exercise and diet—it is important to note that most cytokine concentrations remained within physiological limits. Thus, the observed shifts likely reflect modulation of low-grade systemic inflammation rather than suppression of overt inflammatory activity. In this context, the term *anti-inflammatory effect* refers to attenuation of immune activation rather than normalization of pathological inflammation. Although baseline dispersion suggested a potential for regression toward the mean, the use of a repeated-measures ANOVA focusing on the time × group interaction allowed us to evaluate the intervention effect independently of baseline differences.

In the control group, minor nonsignificant cytokine increases occurred despite stable DMT use. These are interpreted as random fluctuations within physiological variability rather than loss of treatment efficacy.

Third, the lack of objective adherence tracking, absence of subgroup analyses (e.g., by sex, age, or MS subtype), and possible regression-to-the-mean effects should be addressed in future work (nonetheless, differential attrition may have influenced group comparability and is discussed as a potential source of bias, possibly interacting with baseline variability in disability and cytokine levels).

The relatively high dropout rate in the WHM group emphasizes the need for tailored protocols that balance efficacy with patient comfort and adherence. The absence of statistically significant differences in dropout rates may be due to limited power and the unexpectedly high dropout rate in the control group. We suppose that patients in control group had less motivation for second visit and completing the study compared to two “active” interventional groups.

Although adherence to home-based activities was regularly monitored, self-report remains suboptimal; future studies should incorporate objective adherence tools (e.g., wearable sensors or digital logs).

Longer and larger randomized trials are warranted to explore sustained effects on neurodegeneration and disease progression. Integrating personalized WHM and exercise regimens, validated biomarkers, and longitudinal follow-up will be crucial for determining their true disease-modifying potential in multiple sclerosis.

## Conclusion

This pilot study provides preliminary evidence that targeted non-pharmacological interventions may attenuate markers of systemic immune activation in MS. While their mechanisms of action differ, they converge in reducing systemic inflammation, the effect on neurodegeneration is less conclusive, particularly over short intervention periods. Both physical activity and the Wim Hof Method (WHM) might represent complementary therapeutic approaches for slowing down the subtle, chronic progression of MS that occurs despite successful treatment of acute relapses and MRI-visible lesions, likely driven by chronic neuroinflammation.of exercise

By leveraging the unique strengths of exercise and WHM, clinicians and researchers can expand the arsenal of non-pharmacological strategies for MS, ultimately improving patient outcomes and quality of life. Importantly, both interventions were safe and well tolerated, with no intervention-related adverse events.

Both our interventions highlight the need for longitudinal follow-up assessing NfL dynamics and disability progression which will clarify whether early immunomodulation translates into slower neurodegeneration. Integration of personalized protocols, especially for WHM, are crucial to determine optimal duration and int wensity without overexposure to stressors​​.

## References

[1] Giovannoni G et al (2022) Smouldering multiple sclerosis: the ‘real MS’. Ther Adv Neurol Disord 15:17562864211066751 35096143 10.1177/17562864211066751 PMC8793117

[2] Scalfari A et al (2024) Smouldering-associated worsening in multiple sclerosis: an international consensus statement on definition, biology, clinical implications, and future directions. Ann Neurol 96(5):826–845 10.1002/ana.27034 39051525

[3] Carlson AK, Fox RJ, Pathophysiology (2024) Diagnosis, treatment and emerging neurotherapeutic targets for progressive multiple sclerosis. Neurol Clin 42:39–54 37980122 10.1016/j.ncl.2023.07.002

[4] Dalgas U, Langeskov-Christensen M, Stenager E, Riemenschneider M, Hvid LG (2019) Exercise as medicine in multiple Sclerosis-Time for a paradigm shift: Preventive, Symptomatic, and Disease-Modifying aspects and perspectives. Curr Neurol Neurosci Rep 19:88 31720862 10.1007/s11910-019-1002-3

[5] Motl RW, Pilutti LA (2024) Advancements and challenges in exercise training for multiple sclerosis: comprehensive review and future directions for randomized controlled trials. Neurol Ther 13:1559–1569 39271645 10.1007/s40120-024-00656-z PMC11541987

[6] Opara J (2024) The role of lifestyle physical activity in preventing multiple sclerosis. Hum Mov 25:1–9

[7] Du L et al (2024) Effects of exercise in people with multiple sclerosis: a systematic review and meta-analysis. Front Public Health 12:1387658 10.3389/fpubh.2024.1387658 PMC11039920 38660348

[8] Declerck L, Bobitt J, Herman C, Motl RW (2025) Exercise training in multiple sclerosis: Preparing for dissemination and implementation based on integrating the NIH stage model of intervention development. Multiple Scler Relat Disorders 102:106623 10.1016/j.msard.2025.106623 40714727

[9] Gaemelke T, Frandsen JJ, Hvid LG, Dalgas U (2022) Participant characteristics of existing exercise studies in persons with multiple sclerosis – A systematic review identifying literature gaps. Multiple Scler Relat Disorders 68:104198 10.1016/j.msard.2022.104198 36257149

[10] Blázquez-Fernández A, Navarro-López V, Marcos-Antón S, Cano-de-la-Cuerda R (2025) Effects of physical exercise on neurofilament light chain and glial fibrillary acidic protein level in patients with multiple sclerosis: A systematic review and bayesian network Meta-Analysis. JCM 14:839 39941510 10.3390/jcm14030839 PMC11818769

[11] Gravesteijn AS et al (2023) Brain-derived neurotrophic factor, neurofilament light and glial fibrillary acidic protein do not change in response to aerobic training in people with MS-related fatigue – a secondary analysis of a randomized controlled trial. Multiple Scler Relat Disorders 70:104489 10.1016/j.msard.2022.104489 36621163

[12] Ercan Z, Bilek F, Demir CF (2021) The effect of aerobic exercise on neurofilament light chain and glial fibrillary acidic protein level in patients with relapsing remitting type multiple sclerosis. Multiple Scler Relat Disorders 55:103219 10.1016/j.msard.2021.103219 34433118

[13] Joisten N et al (2021) Exercise diminishes plasma neurofilament light chain and reroutes the kynurenine pathway in multiple sclerosis. Neurol Neuroimmunol Neuroinflamm 8:e982 33782190 10.1212/NXI.0000000000000982 PMC8054957

[14] Kox M et al (2014) Voluntary activation of the sympathetic nervous system and Attenuation of the innate immune response in humans. Proc Natl Acad Sci U S A 111:7379–7384 24799686 10.1073/pnas.1322174111 PMC4034215

[15] Zwaag J, Naaktgeboren R, van Herwaarden AE, Pickkers P, Kox M (2022) The effects of cold exposure training and a breathing exercise on the inflammatory response in humans: A pilot study. Psychosom Med 84:457–467 35213875 10.1097/PSY.0000000000001065 PMC9071023

[16] Buijze GA et al (2019) An add-on training program involving breathing exercises, cold exposure, and meditation attenuates inflammation and disease activity in axial spondyloarthritis - A proof of concept trial. PLoS ONE 14:e0225749 31790484 10.1371/journal.pone.0225749 PMC6886760

[17] Adamová LM (2024) Impact of dance classes on motor and cognitive functions and gut microbiota composition in multiple sclerosis patients: randomized controlled trial. Eur J Sport Sci 24:1186 38967986 10.1002/ejsc.12166 PMC11295098

[18] Guo LY, Lozinski B, Yong VW (2020) Exercise in multiple sclerosis and its models: focus on the central nervous system outcomes. J Neurosci Res 98:509–523 31486115 10.1002/jnr.24524

[19] Lozinski BM, Yong VW (2022) Exercise and the brain in multiple sclerosis. Mult Scler 28:1167–1172 33124511 10.1177/1352458520969099

[20] Proschinger S et al (2025) Sportizumab – Multimodal progressive exercise over 10 weeks decreases Th17 frequency and CD49d expression on CD8 + T cells in relapsing-remitting multiple sclerosis: A randomized controlled trial. Brain Behav Immun 124:397–408 39675643 10.1016/j.bbi.2024.12.017

[21] Zong B et al (2023) Mechanisms underlying the beneficial effects of physical exercise on multiple sclerosis: focus on immune cells. Front Immunol 14:1260663 10.3389/fimmu.2023.1260663 PMC10570846 37841264

[22] Buijze GA et al (2019) An add-on training program involving breathing exercises, cold exposure, and meditation attenuates inflammation and disease activity in axial spondyloarthritis – A proof of concept trial. PLoS ONE 14:e0225749 31790484 10.1371/journal.pone.0225749 PMC6886760

[23] Kaymaz K, Beikler T (2019) Th17 cells and the IL-23/IL-17 axis in the pathogenesis of periodontitis and Immune-Mediated inflammatory diseases. IJMS 20:3394 31295952 10.3390/ijms20143394 PMC6679067

[24] Zheng M-Y, Luo L-Z (2025) The role of IL-17A in mediating inflammatory responses and progression of neurodegenerative diseases. IJMS 26:2505 40141149 10.3390/ijms26062505 PMC11941770

[25] Mado H, Stasiniewicz A, Adamczyk-Sowa M, Sowa P (2024) Selected interleukins relevant to multiple sclerosis: new Directions, potential targets and therapeutic perspectives. IJMS 25:10931 39456713 10.3390/ijms252010931 PMC11506881

